# CLINICOPATHOLOGICAL EVALUATION OF RADIATION INDUCED BASAL CELL CARCINOMA

**DOI:** 10.4103/0019-5154.43222

**Published:** 2008

**Authors:** Naser Tayyebi Meibodi, Masood Maleki, Zari Javidi, Yalda Nahidi

**Affiliations:** *From the Department of Pathology of Mashhad University of Medical Sciences, Mashhad, Iran*; 1 *From the Department of Dermatology of Mashhad University of Medical Sciences, Mashhad, Iran*

**Keywords:** *Basal cell carcinoma*, *basal cell epithelioma*, *radiotherapy*

## Abstract

**Background:**

Development of skin neoplasms is one of the most important chronic complications of radiation therapy. Basal cell carcinoma (BCC) is the most frequent carcinoma occurring at the region of the body to which radiotherapy was delivered.

**Aim:**

The aim of this study was to evaluate clinical and histological aspects of basal cell carcinoma in patients with a history of radiotherapy.

**Materials and Methods:**

Medical records and microscopic slides of 80 patients with basal cell carcinoma who had received radiotherapy (1996-2006) were reviewed in pathology department of Imam Reza hospital of Mashhad, Iran. Collected data were analyzed statistically using descriptive test.

**Results:**

60 men and 20 women were included, majority of them in their sixties. Plaque was the most common clinical pattern of basal cell carcinoma. Fifty one percent of the patients had pigmented and 42.5% had multiple lesions. Scalp was the most common site of involvement. Histologically, macronodular and pigmented carcinoma were the most predominant forms of basal cell carcinoma.

**Discussion:**

Majority of patients had scalp involvement and multiple lesions. Nodular and pigmented forms were the most common histological findings. We suggest the need for close supervision in patients with a history of radio therapy in the past.

## Introduction

Evidence that ionizing radiation is carcinogenic first came from reports of nonmelanoma skin cancers on the hands of workers using early radiation devices. An increase in relative risk of basal cell carcinoma has also been found among uranium workers, radiologists and children irradiated for an enlarged thymus gland or tinea capitis.^1^

Radiotherapy has acute and chronic damages on skin. Chronic radiation damage has common manifestations such as telengiectasia, irregular hyperpigmentation, atrophy, and scar formation. It is well known that radiation-induced skin cancer may appear at sites of prior radiation, even if there is no evidence of chronic radiation damage, as in the present case. Several studies reported in the last decade also supported the issue that variable factors such as anatomic location, radiation type and dosage, age at exposure, post-treatment time, sun exposure, and ethnic and genetic factors have considerable effects on the incidence and type of skin cancers in the irradiated population.^2^

Skin neoplasms especially basal cell carcinoma (BCC) and then squamous cell carcinoma (SCC) are the main complications of radiotherapy.^3^ Incubation periods of post-radiation skin malignancies are very long. Skin cancer is known as a late complication of ionizing radiation.^3^,^4^ Radiation-induced skin cancer may develop any time from 2 to 65 years after exposure, with an estimated median latency of 20 to 45 years.^2^,^4^–^7^ Total dose of radiation and irradiated site exposed to sunlight can lead to short incubation period.^6^ BCC occurs in irradiated areas usually in elderly and less frequently at young ages. Incubation period between exposure to ionizing radiation and BCC symptoms is shorter in young patients.^8^,^9^ Radiation exposures to the scalp during childhood for tinea capitis were associated with a four-fold increase in skin cancer, primarily basal cell carcinomas, and a three-fold increase in benign skin tumors.^2^,^10^

Relative risk of BCC is higher in patients who had received low dose radiation to head and neck area.^2^

This study is performed to evaluate clinical and histological aspects of basal cell carcinoma in patients with a history of radiation.

## Materials and Methods

This study was carried out at the dermatology ward of Imam-Reza hospital of Mashhad. During a 10-year period (1996-2006), a total of 648 cases of BCC were admitted. Regarding to their medical records, 80 cases were selected. All of them had a history of radiotherapy for treatment of tinea capitis during childhood. The files of all patients were collected and the following data were recorded: age, gender, clinical form, number and size of lesions, age at onset of radiation and sample number. Parameters such as ulcer, histologic pattern and pigmentation were reviewed using microscopic slides.

Collected data were analyzed statistically by SPSS software (version 13) using *qui-square*, T-student and Man-Whitney tests with 95% confidence interval.

## Results

Among 80 patients with positive history of radiation therapy, 60 (75%) were men and 20 (25%) were women (male: female ratio was 3:1). Predominantly, BCC had been developed in 5^th^ (28/75%), 6^th^ (42.5%) and 7^th^ (18.75%) decades of age. Average age of our cases was 56 years.

Plaques (74%), papules (13%), nodules (10%) and patches (3%) were the most common clinical patterns of basal cell carcinoma. Fifty one percent of the patients had pigmented lesions and clinical ulcer was detected in 53%. Forty six cases had only one lesion but 18 cases had two, 8 cases had three and 8 cases had five lesions. The mean number of lesions was 1.83 per patient. Scalp (71%), forehead (6%), nose (5%) and periorbital area (5%) were the most common sites of involvement.

Histologically, nodulocystic (60%) and micronodular (17.5%) carcinoma were the most predominant forms of BCC. 37.5% had ulcers and 72% had pigmentation.

## Discussion

Different types of neoplasms such as skin cancers, angiosarcoma, malignant fibrous histiocytoma, thyroid carcinoma and brain tumors may occur following radiotherapy.^2^,^4^ BCC (70-100%)^11^ and then SCC are the most common malignancies in patients receiving radiation.^3^,^4^

Karagas suggests that exposure to therapeutic radiation is associated with BCC but not with SCC.^1^ These neoplasms usually occurs after radiation therapy for treatment of tinea capitis, hypertrophic tonsillitis^4^ acne vulgaris,^1^,^4^ atopic dermatitis and hyperthyroidism^4^ and rarely following cancer of cervix and nasopharynx,^12^ neck lymph node tuberculosis,^5^ medulloblastoma,^13^ ankylosing spondylitis,^14^ hemangioma^15^,^16^ and hirsutism.^7^ A recent report suggested an increased risk of nonmelanoma skin cancer among the latest Japanese atomic bomb survivors.^1^

Arai *et al*, defined radiation-associated second cancer using the following criteria:^1^ difference of histologic type from the primary cancer^2^ latency period of at least 2 years after radiation therapy and^3^ development site in the irradiated field.^4^

Regarding to Maalej and colleagues report the average latency period between irradiation and cancer occurrence was 36 ± 14 years.^3^ Meseddi's report suggests that the interval between irradiation and the onset of carcinoma varied between 21 and 51 years.^11^

The time between receiving radiation therapy and onset of BCC was 30 to 70 years in our cases. The average patients' age at the time of irradiation was 12 ± 6 years and 5 to 17 years in Maalej's and Mseddi's studies respectively.^3^,^11^

Majority of our cases were irradiated at less than 12 years of age. Among all 648 patients suffering from BCC, 80 cases (12.5%) had a history of radiation therapy. This was 41% in Akhyani's study^17^ which can be explained by the professional aspects of Razi hospital and also division of our patients in 2 major dermatologic centers in Mashhad.

Ron and colleagues reported that the average radiation dose for treatment of tinea capitis was 7GY. The relative risk of radiogenic skin cancer did not differ significantly between men or women or by time since exposure but was greatest following exposures in early childhood. The estimated excess relative risk was 0.7 per Gy.^10^

Shore's study showed a relative risk of 3.6 (95% confidence interval, 2.3-5.9) for BCC of the head and neck among irradiated Caucasians in response to a dose of about 4.8Gy to scalp. Children irradiated at young ages had the highest BCC risk. He also mentioned that low ages at the time of exposure and UV radiation increase the relative risk of BCC.^18^

Radiation induced BCC usually occurs with low to moderate doses of radiation for treatment of tinea capitis, hypertrophic tonsillitis, acne vulgaris, atopic dermatitis and hyperthyroidism. But SCC seems to develop after high-dose radiation.^2^,^4^,^7^

In 1987 Vloten *et al*, examined 605 patients treated by irradiation for benign diseases in the head and neck region. They reported 30 skin tumors mainly BCC and mentioned that severity of radiodermatitis is associated with a higher prevalence of skin cancer and the number of radiation-induced skin cancers rises with the post-treatment time.^2^

The mean age of our cases was 56 years (ranged 40 to 80) that is higher than Maalej's cases in which patient's age at diagnosis of malignancy varied from 20 to 83 years with an average of 47 years.^3^

Among 98 patients of Maalej's study, in 61 patients (62%), the scalp appeared normal and in 38% radio dermatitis was noted. Basal cell carcinomas were the most frequent tumors arising on chronic radiodermatitis.^3^ The radiogenic BCCs tend to be multiple and they manifest as conventional nodular BCC, superficial BCC and rarely, fibroepithelioma Pinkus and keloidal types.^7^ Also, linear BCC can develop rarely in the irradiated areas.^19^

Majority of our cases were men (75%) similar to Mseddi's study.^11^ It can be due to higher prevalence of tinea in leading to higher radiation therapy in men. Moreover, occupation and sunlight exposure are also important risk factors. In Mseddi's study 40% of BCC occurred on the occipital area and 65% were in the scalp site. The number of lesions varied from 1 to 5 and the size from 2 to 45 mm.^11^ About 41% of Akhyani's cases had a positive history of irradiation. This rate was 90% in patients with BCC on head, ear helices and neck. Also number of lesions was higher in cases having received previous radiation.^17^

Scalp was the main area of involvement in our cases (71%) and 42.5% had multiple lesions varying from 3 mm to 8 × 4 cm.

Also Shore reported several cancers in irradiated patients.^9^,^18^ About 40% of his irradiated cases have had multiple BCCs.^18^ The average of lesions number was 1.8 per patient in our study, 1.5 per patient in Maalej's study and 1.76 per patients in Mseddi's study.^3^,^11^ In Mseddi's study, nodular BCC and pigmentation was detected in 51% 64% of cases clinically but histological study showed a nodular pattern in 76% and pigmentation in 63% of cases.^11^ Despite of Akhyani's report in which noduloulcerative BCC was the main clinical from (30.5%),^17^ the most common forms in our study were plaque (74%) and pigmented (51%). The most frequent histological type in both Akhyani's and Malekzadeh's studies was solid tumors (53.5% and 30%).^17^,^20^ Microscopic study revealed 72% pigmentation in our study which was higher than Mseddi's (62%)^11^ and Malekzadeh's (44%) reports.^20^

## Conclusion

Periodical lifetime examination of irradiated areas in addition to scalp and ear helices is indicated for patients with a history of radiation. Moreover, it is very important to create awareness in patients of referring to their physician for any suspicious lesions.
